# Groundwater quality trend and trend reversal assessment in the European Water Framework Directive context: an example with nitrates in Italy

**DOI:** 10.1007/s11356-020-11998-0

**Published:** 2021-01-07

**Authors:** Eleonora Frollini, Elisabetta Preziosi, Nicoletta Calace, Maurizio Guerra, Nicolas Guyennon, Marco Marcaccio, Stefano Menichetti, Emanuele Romano, Stefano Ghergo

**Affiliations:** 1 IRSA-CNR, Via Salaria km 29,300, 00015 Monterotondo Roma, Italy; 2 ISPRA, Via Vitaliano Brancati, 48, 00144 Rome, Italy; 3 ARPAE Emilia Romagna, Largo Caduti del Lavoro, 6, 40122 Bologna, Italy; 4 ARPAT Toscana, Via N. Porpora, 22, 50144 Firenze, Italy

**Keywords:** Groundwater chemical status, Ground Water Directive, River Basin Management Plans, Mann-Kendall, Pettitt test, Nitrate pollution

## Abstract

Groundwater resources are of utmost importance in sustaining water related ecosystems, including humans. The long-lasting impacts from anthropogenic activities require early actions, owing to the natural time lag in groundwater formation and renewal. The European Union (EU) policy, within the implementation of the Water Framework Directive (WFD), requires Member States to identify and reverse any significant and sustained upward trend in the concentration of pollutants, defining specific protection measures to be included in the River Basin Management Plans (RBMP). In Italy, official guidelines for trend and trend reversal assessment have been published recently. Statistical methods, such as the Mann-Kendall test for trend analysis and the Sen’s method for estimating concentration scenarios, should be applied at the fixed terms stated by the WFD implementation cycles to identify upward trends, while the Pettitt test is proposed for the identification of trend reversal. In this paper, we present an application of a slightly modified version of the Italian Guidelines to a groundwater body in Northern Italy featuring nitrate pollution and discuss its advantages and limitations. In addition to Pettitt test, for the trend reversal analysis, we apply the Mann-Kendall test in two sections and compare the results. We conclude that this method seems more reliable than Pettitt test to identify a reversal point in quality time series. The overall procedure can be easily applied to any groundwater body defined at risk across Europe, for the assessment of the upward trends of pollutants and their reversal, even with little chemical monitoring data. Although focused on the EU legislative framework, this procedure may be relevant for a wider context, allowing to individuate upward trend as early warning for contamination processes in an integrated water resources management context.

**Supplementary Information:**

The online version contains supplementary material available at 10.1007/s11356-020-11998-0.

## Introduction

Groundwater plays an important role in drinking water supply: about 50% of the world’s human consumption is sustained by groundwater, being the primary source for 1.5–2.8 billion people (Giordano 2009). In Europe, the share of groundwater needed nationally to meet the total demand for freshwater ranges from 9% up to 100% (Scheidleder et al. 1999), and in Italy, more than 85% of the drinking water is supplied by aquifer exploitation from wells and springs (Onorati et al. 2006; ISTAT 2017). Further, groundwater provides the base flow for many surface water systems; therefore, its qualitative and/or quantitative degradation may deteriorate the status of the surface waters, eventually jeopardizing the associated aquatic ecosystems and directly dependent terrestrial ecosystems (Griebler et al. 2019; Pastor et al. 2019; Qiu et al. 2019). Since groundwater moves slowly through the aquifers, the effects of human activities can appear much later than the harmful event and last for decades or even longer, threatening the uses of groundwater for many years. Indeed, it may be difficult to reclaim groundwater even after the source of pollution is removed.

The quality of groundwater is threatened by a multitude of processes, both diffuse, such as leaching of nitrate and pesticides from cultivated land, and localized (chemical waste deposits, landfills, oil tanks and contaminated sites). In order to preserve and/or improve the quality of groundwater, the assessment of the chemical status and of upward trends of pollutants is fundamental for the identification of those groundwater bodies (GWB) in which protective measures are most needed. Furthermore, since the positive effects of actions to improve the quality of groundwater can be detected after some years from their implementation, it is necessary to identify upward trends of the pollutants in advance (Craig and Daly 2010).

The identification, monitoring and assessment of the chemical status of groundwater and any significant and sustained upward trend in the concentration of any pollutant are required in Europe by the Water Framework Directive (WFD, 2000/60/EC) and the Groundwater Directive (GWD, 2006/118/EC) (Urresti-Estala et al. 2016). The latter, in Italy, has been implemented into the national Legislative Decree 30/2009. The GWD defines a significant upward trend “any statistically and environmentally significant increase of concentration of a pollutant, group of pollutants, or indicator of pollution in groundwater for which trend reversal is identified as being necessary”, because they “present a significant risk of harm to the quality of aquatic ecosystems or terrestrial ecosystems, to human health, or to actual or potential legitimate uses of the water environment”. The European Commission (2009) specifies that the statistical significance of trends must be verified using a recognised statistical trend assessment technique, without no further indication on which “recognised” technique should be applied (Urresti-Estala et al. 2016). To this end, some Member States use the parametric test ANOVA and the non-parametric Mann-Kendall test (Grima et al. 2015; Gourcy et al. 2019). In addition, if any upward trend can lead to the failure to meet one or more of the environmental objectives of the WFD, this trend is also significant from the environmental point of view (European Commission 2009). According to some Member States, this happens when the projected trend line exceeds the threshold value (TV) for that pollutant in the two subsequent River Basin Management Plan (RBMP) cycles, that is, until 2027 or when the trend line exceeds the 75% of the TV, which is the starting point for trend reversal, in the immediately subsequent RBMP cycle (GWD). Others, such as France, consider a trend as environmentally significant when 40% of the TV would be reached at the end of the WFD cycle, should the trend continue with that slope (Gourcy et al. 2019).

Trend analyses of water quality time series have important implications for pollution control and environmental decision-making, and many examples of application or methodological reviews have been published so far, especially for stream water quality analysis. Methods commonly applied are mainly traditional statistical methods including parametric (i.e. linear regression, polynomial regression) and nonparametric (e.g. Mann Kendall) methods (Hirsch et al. 1991; Esterby 1996; Lopez et al. 2015; Urresti-Estala et al. 2016; Huang et al. 2017). Whatever the method adopted to test “statistical” and/or “environmental” significance, it is worth noting that time series of water quality data might be influenced by changes in sampling and laboratory practices. Wahlin and Grimvall (2008) found strong evidence that long-term trends in measured nutrient concentrations in surface waters can be more extensively influenced by changes in sampling and laboratory practices than by actual changes in the state of the environment. Although an analysis of measure uncertainty in groundwater monitoring is outside the scope of this paper, regular retrospective analyses and joint analysis of several time series of data should be applied before undertaking trend detection.

According to the EU regulation, Member States are required to take appropriate actions to reverse these trends through the application of a programme of measures (PoMs) in the framework of the RBMP, to ensure that there will not be future failures to meet the environmental objectives for the groundwater body. At the same time, WFD requires Member States to undertake a cost-effectiveness analysis of PoMs (Martin-Ortega 2012).

In Europe, 74% of groundwater bodies (by area) has a good chemical status, 25% has a poor chemical status and 1% has not been classified (EEA 2018). Although 160 different chemicals have been reported as causing poor chemical status, the reason for failure is due mainly to nitrates and pesticides, witnessing that agriculture is still a main issue (although other sources for nitrates should not be neglected), then to ammonium, sulphates and chlorides (related to seawater intrusion). Among these pollutants, significant and sustained upward trends were identified mainly for nitrates, chloride, pesticides and sulphate, in 19 Member States out of 25 examined by the European Environmental Agency (EEA), involving 9.9% of total GWB area. In contrast, 14 Member States reported trend reversals in 5.9% of GWB area mainly for nitrates, ammonium, sulphates and chlorides (EEA 2018).

In Italy, 57.7% (by area) of groundwater bodies were classified in good chemical status, 34.4% in poor status and 7.9% still have not been classified (data available until 2015, ISPRA 2017). The chemical parameters that cause the poor chemical status are mainly not only inorganic compounds (such as nitrate, sulphate, fluoride, chloride, boron, metals) but also chlorinated compounds, aromatic compounds and pesticides. Due to the current short length of the chemical time series, the assessment of significant upward trends in Italy will be available only at the end of the current RBMP in 2021 (ISPRA 2017).

Recently, national guidelines have been published in Italy (ISPRA-CNR.IRSA 2017), reporting a detailed methodology to assess upward trends of pollutants and their reversal.

In this paper, we describe the methodological approach proposed by the Guidelines (“Materials and methods”) and present an application of this procedure, slightly modified, to a groundwater body in Emilia-Romagna (Po Plain, Northern Italy) (“Results”). Finally, we discuss advantages and disadvantages in relation with the approaches proposed by other European Member States (“Discussion”).

## Materials and methods

### The Italian Guidelines: when and how to assess an upward trend and a trend reversal

**Fig. 1:**
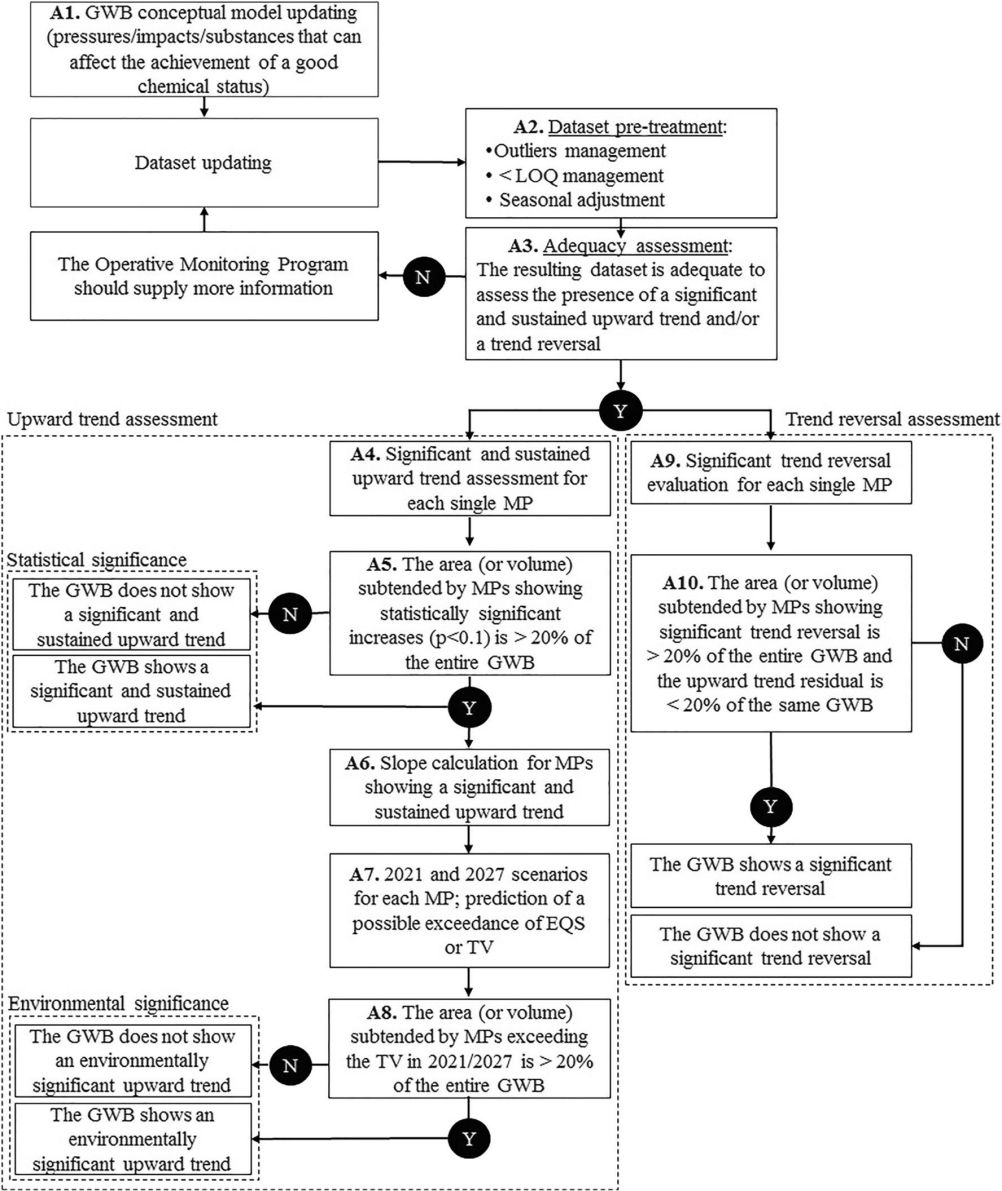
Main procedure for the evaluation of upward trend and trend reversal for GWB defined “at risk”. Y, yes; N, no

Following the GWD, the Italian legislation (Decree 30/2009) requires to perform a trend assessment for those compounds/parameters that put the groundwater body at risk of not achieving the WFD environmental objectives, or are somehow jeopardizing the achievement and maintenance of good chemical status. The procedure provided by the Guidelines (Fig. 1) deals with the several steps needed to produce a robust output, from the conceptual model update (Fig. 1, box A1), to data processing before statistical analysis (Fig. 1, box A2), to the calculation of the trend (Fig. 1, boxes A4-A8) or trend reversal at the GWB scale (Fig. 1, boxes A9 and A10). The Guidelines suggest that all the available data from the monitoring activities should be considered (i.e. both surveillance and operational monitoring) to produce or update a robust conceptual model of the GWB. Analytical values below the LOQ (limit of quantification) should be set to half of the highest quantification limit occurring in the time series (Grath et al. 2001; GWD, Annex 4). In addition, to avoid bias due to possible dependence of the concentration on seasonality, it is suggested to average all the data on an annual basis, else to choose one seasonal value per year. The conceptual model plays a key role in providing the necessary knowledge for a correct decision.

Adequacy of the data set for the trend assessment (Fig. 1, box A3) is ensured when at least 8 (annual) values are available, for a time span of observations ranging from 8 to 15 years (Grath et al. 2001). Data older than 15 years prior to the time of the analysis are not admitted, to avoid relying on data that are too old and do not represent the current state of the GWB (Grath et al. 2001). Similarly, time series with the most recent data dating back more than 3 years prior to the assessment should be discarded to prevent the assessment of past situations. Time series with missing years can be used as long as there is no missing data for two or more consecutive years.

The procedure for trend calculation is applied first to each monitoring point (MP) (Fig. 1, box A4) and then to the whole groundwater body (Fig. 1, box A5) as explained in the following paragraph.

### How to define a statistically and environmentally significant trend

In statistics, significance is defined as the probability to reject the null hypothesis we are testing. Applying this concept to groundwater chemical time series, the null hypothesis is whether there is no significant monotonic trend of a pollutant. The Mann-Kendall test (Mann 1945; Kendall 1975) is a very popular one, used to statistically assess whether there is a monotonic upward or downward trend over time. It is a non-parametric test; hence, it does not require assumptions about the probability distribution of the dataset, and it is commonly used for the statistical treatment of environmental data (Ducci et al. 2019; Helsel and Frans 2006; Urresti-Estala et al. 2016; Zhang et al. 2006). Hence, it can be applied to time series that do not follow the normal distribution, such as groundwater chemical data, which generally have asymmetric or non-normal distributions (UKTAG 2012). Further, it is robust against the influence of possible outliers and appropriate for seasonally corrected data (Amirataee and Zeinalzadeh 2016; Oliva et al. 2016; Salmi et al. 2002; Visser et al. 2009). Finally, because Mann-Kendall test is based on ranking, it can be computed also in the presence of missing data or with < LOQ data, although the performance of the test might be affected. The Guidelines propose a threshold of 90% for the assessment of statistical significance.

The dependence of the statistical significance on the sample size was taken into consideration according to Hollander and Wolfe (1973). This is a key point, as in these situations one has to deal with a limited number of temporal observations (< 40 annual data). For this reason, the S statistics from Hollander et al. (2014); Hollander and Wolfe (1973) and Kendall (1975) for the one-tail test was implemented in a spreadsheet (described in Appendix A and provided as supplementary material).

The identification of significant and sustained upward trends at the MP level is performed by applying the one tail Mann-Kendall test (MK test) to the processed time series of the selected pollutants. The MK test calculates the S statistics as follows:

1 $$ S={\sum}_{i=1}^{n-1}{\sum}_{j=i+1}^n\operatorname{sign}\ \left({x}_i-{x}_j\right) $$

where *n* is the total number of observations, *x*_*i*_ and *x*_*j*_ are two generic sequential data values and the function sign(*x*_*i*_ *− x*_*j*_) assumes the following values:

2 $$ \operatorname{sign}\ \left({x}_i-{x}_j\right)=\left\{\begin{array}{c}+1\kern0.5em \mathrm{if}\ {x}_i>{x}_j\\ {}0\ \mathrm{if}\ {x}_i={x}_j\\ {}-1\ \mathrm{if}\ {x}_i<{x}_j\end{array}\right. $$

When the MK test rejects the null hypothesis at the 90% confidence level (which can be calculated using the Hollander and Wolfe (1973) table implemented in the spreadsheet in the supplementary material), the upward trend of the pollutant is considered statistically significant at that monitoring point (Fig. 1, box A4).

To evaluate if the GWB is subject to a significant upward trend, a spatial assessment of the trend identified at one or more MPs is performed as follows. The Guidelines suggest two different methods to calculate the percentage of area or volume of GWB to be assigned to each MP. If sufficient knowledge of the GWB is available, each MP is assigned a specific extent/volume based on the conceptual model of the aquifer (e.g. based on groundwater flow models, tracer test results); otherwise, each MP is assigned the same percentage of GWB extension/volume. The latter is by far the most frequently applied method. If more than 20% of the entire area or volume of the GWB shows a statistically significant trend, that GWB is considered to be subject to a statistically significant upward trend for that pollutant (Fig. 1, box A5).

The second question to ask is whether this trend is significant from an environmental point of view. The Guidelines clarify that the environmental significance of the increase means a growth in the values over time with a rate such as to jeopardize the achievement of environmental objectives for that groundwater body (European Commission 2009). Following the Guidelines, an upward trend is environmentally significant at the MP level when its extrapolation exceeds the starting point for trend reversal (that is, 75% of the quality standard or threshold value of the substance) or the quality standard/threshold value itself, putting that GWB at risk of not achieving the WFD environmental objectives. At the GWB scale, the trend is environmentally significant when the MPs with environmentally significant trend represent more than 20% of the entire area/volume of GWB (Fig. 1, box A8). If this is the case, the upward trend has to be reversed through the application of appropriate measures (GWD).

To carry out this evaluation, the scenarios in 2021 and/or 2027 (representing the end of the 2nd and 3rd RBMP cycles) are examined. For this purpose, Sen’s slope non-parametric method (Sen 1968), which robustly handles outliers and gaps in the time series (Salmi et al. 2002; UKTAG 2012), is applied to calculate the slope of the trend and extrapolate it into the future (Fig. 1, boxes A6, A7).

Sen’s method provides a nonparametric estimate of the slope *d* of a time series by considering the *k* pairs for which *j* is greater than *i*, where *x*_*i*_ is the pollutant concentration at time *i*. For each couple of values (*x*_*i*_, *x*_*j*_) the slope *d*_*k*_ is calculated as follows:

3 $$ {d}_k=\frac{x_j-{x}_i}{j-i} $$

Sen’s estimator of slope is the median of the *k* values of *d*_*k*_.

The intercept (*m*_*t*_) is obtained as the median of the intercepts of each couple of values (*x*_*i*_, *x*_*j*_) calculated as follows:

4 $$ {m}_t=\frac{j\cdot {x}_i-i\cdot {x}_j}{j-i} $$

As per the Guidelines, the scenarios at 2021 and/or 2027 are calculated adding to the last observed concentration value the total increase in concentration, given by slope of the trend multiplied by the number of years between 2021/2027 and the last observation year.

Differently in this paper, the scenarios are calculated as the projection at year 2021/2027 of the Sen’s slope calculated with Eqs. 3 and 4.

### Trend reversal using the Pettitt test

The trend reversal analysis aims at statistically demonstrating that an upward trend has been reversed, as requested by the GWD. Therefore, the reversal test should be applied to those monitoring sites and pollutants for which a significant and sustained upward trend has been ascertained (Fig. 1, box A9). To this aim, while the MK test was previously applied to test the significance of a monotonic upward trend, the detection of an abrupt change indicating a possible reversal at a given time needs a different statistical tool. The Guidelines propose the use of the non-parametric Pettitt test (Pettitt 1979), able to identify the existence of a changing point in the dataset. The null hypothesis of Pettitt test is that there is no change in the time series while the alternative hypothesis is that there is a shift in the central tendency. If a break in the trend occurs in the year *K*, then the absolute value of the Pettitt statistics (*K*_*t*_) is maximum for that year, and the probable change-point is located where the Pettitt statistics has its maximum.

The Pettitt statistics is defined as follows:

5 $$ {K}_t=\max \mid {U}_{t,T}\mid $$

where

6 $$ {U}_{t,T}={\sum}_{i=1}^t{\sum}_{j=t+1}^T\operatorname{sign}\ \left({x}_i-{x}_j\right) $$

At least 14 annual data are required by the Guidelines to apply this test. As in the upward trend identification, also for the reversal demonstration, some missing data in the time series are acceptable; however, datasets with missing data for two or more consecutive years or with the latest data older than 3 years prior to the assessment should be discarded. Similarly to the upward trend assessment, also the trend reversal is considered significant at the GWB scale if demonstrated on more than 20% of the total area/volume. In addition, if there are still other MPs with an upward trend, they must not exceed 20% of the area/volume of the same GWB (Fig. 1, box A10).

### Trend reversal using the MK test

Differently from the Guidelines, in this case study, we also applied the Mann-Kendall test to the reversal verification, by calculating the S statistics separately on two consecutive sub-sets of the time series (hereinafter referred to as “Mann-Kendall two-section test”). The MK test is applied iteratively to the time series divided into two sections, using the first part of the data set (starting with 8 data and increasing by one at each iteration) for the ascending section and the remaining data for second section. The test identifies a reversal point in the time series when the null hypothesis is rejected at 90% confidence level on the first section with an upward trend and on the second section with a downward trend. In this case, the time series is considered reversed and the starting point of the second section is the reversal year. Because at least 8 data are required for each section, for the Mann-Kendall two-section test, we need at least 15 annual data (in this case, the reversal point is shared between the two sections).

### The “Conoide Trebbia Luretta” groundwater body

**Fig. 2:**
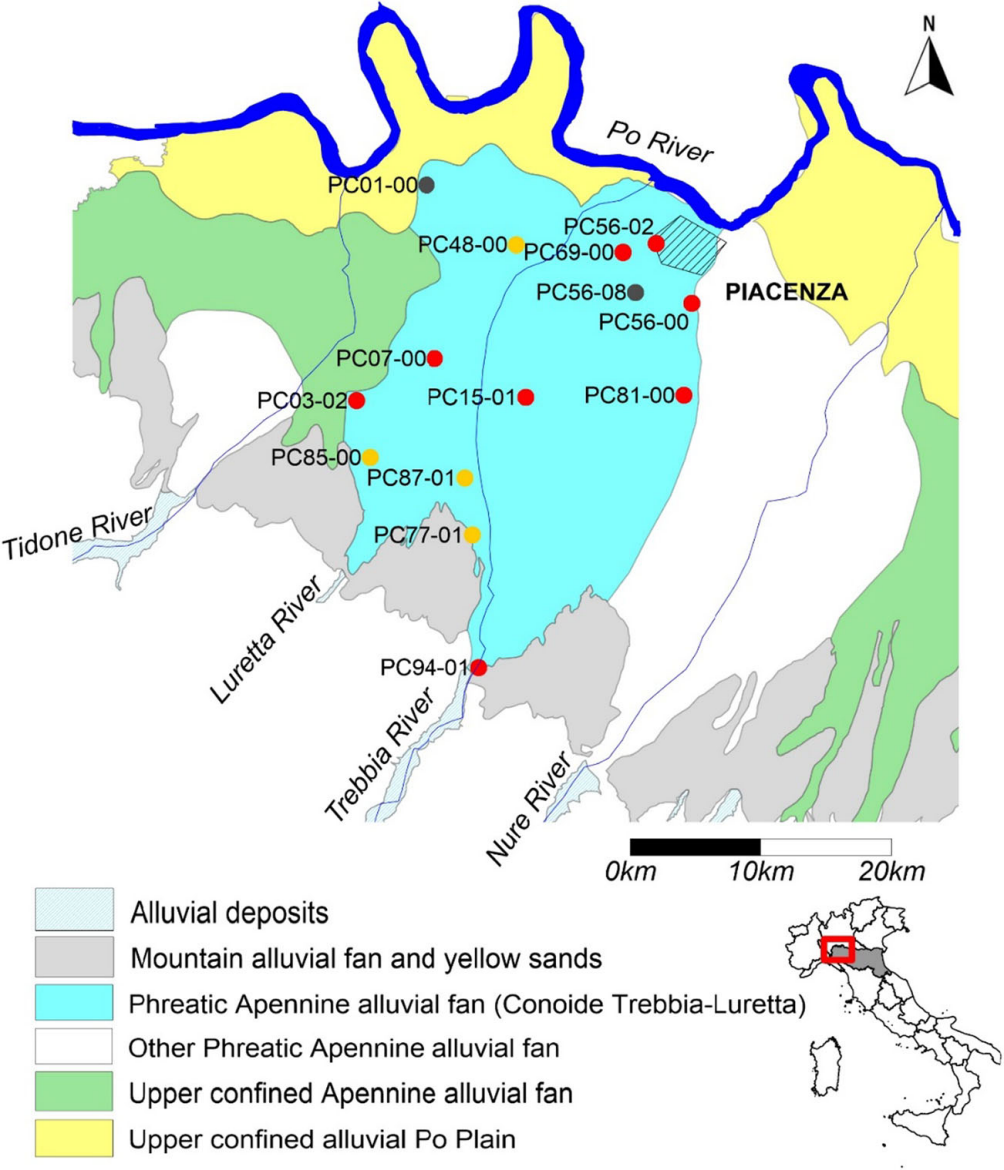
Study area schematic map. Red dots are MPs with statistically significant upward trend; orange dots are MPs with a not significant upward trend; black dots are MPs with a not assessable trend. The light blue area is the Conoide Trebbia-Luretta GWB, and the hatched area is the Piacenza urban area

The “Conoide Trebbia-Luretta” groundwater body is a monolayer phreatic aquifer of the Apennines alluvial fan hydrogeological complex. It is located in Emilia-Romagna Region, on the southern (hydrographic right) bank of the Po River. *Conoide Trebbia-Luretta* is the largest alluvial fan of Emilia-Romagna Region with 184.66 km^2^ of surface (Regione Emilia-Romagna 2010). The hydrogeological flux is direct from South-West to North-East, towards the Po River and the city of Piacenza. The hydrogeological recharge is due to direct input by the rainfall and to the losses of the Trebbia and Luretta rivers. The main anthropogenic pressures on the GWB are the numerous industrial and agricultural activities that cause the failure of the chemical status due to nitrate and chlorinate compounds, whose concentration exceeds the quality standards. Its chemical status is monitored at 14 sampling points (monitoring density 13.2 km^2^) (Fig. 2). The analysed time series extended from 2001 to 2018. All data are available at the ARPAE Emilia-Romagna web site (ARPAE 2020).

## Results

During the observation period, in several years, the quality standard for nitrates (50 mg/L) was exceeded at 3 out of 14 monitoring points (PC01-00, PC56-08 and PC81-00), corresponding to 21.4% of the total GWB area assuming that each of the 14 monitoring point equally represents the groundwater body (Tab. S1).

**Fig. 3:**
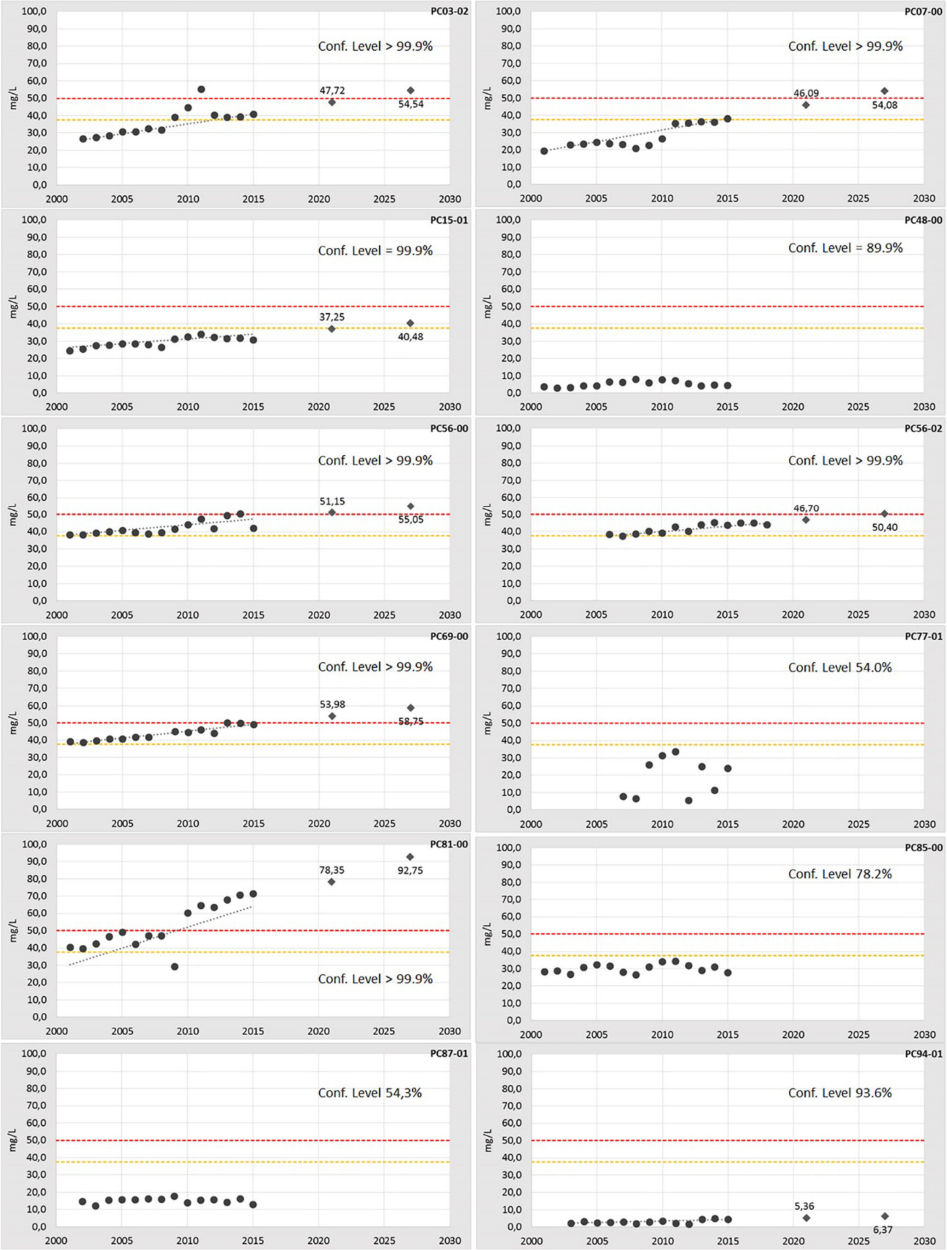
Trend analysis for the “Conoide Trebbia-Luretta” groundwater body. In the charts, black dots represent monitoring data; grey diamonds represent scenarios at 2021 and 2027 for the MPs with a confidence level > 90%. The grey dotted line represents the median slope of the trend, calculated according to the Sen’s method; the intercept is the median of all the intercepts. The red line and the orange line represent the quality standard and the 75% of the quality standard for nitrates, respectively.

The trend assessment was performed with the MK test at the end of the 1st RBMP cycle (2015) using all the previous available data. Two out of 14 MPs (PC01-00 and PC56-08) were excluded due to missing data in their time series. Of the 12 remaining monitoring points, eight (red dots in Fig. 2) show a statistically significant upward trend for nitrates (Fig. 3; Table 1). As suggested by the Guidelines, we assigned the same percentage by weight to each MP because a more detailed conceptual model of the aquifer is not available. By doing so, we assume that each of the 12 monitoring points equally represents the groundwater body (relative weight 8.3%), and the eight points all together represent 66.7% of the whole GWB. Consequently, a significant and sustained upward trend for nitrate can be assumed at the groundwater body scale too, and the evaluation of the scenarios in 2021 and 2027 is therefore needed. The scenarios were elaborated extending in the future the Sen’s slope of each time series with a confidence level above 90% (Fig. 3; Table 1). The results show that the groundwater body would exceed the starting point for trend reversal for nitrate (37.5 mg/L that is the 75% of the quality standard) at 6 MPs in 2021 and the quality standard itself for nitrates (50 mg/L) at the same 6 MPs in 2027, representing 50% of its area, unless adequate measures are implemented. Therefore, the nitrate upward trend for the GWB of the “Conoide Trebbia-Luretta” results “environmentally significant”; as required by WFD, it should be reversed by applying relevant measures during the next cycle and the reversal should be statistically demonstrated.

**Table 1:** Trend assessment by Mann-Kendall test at the end of the 1st RBMP cycle (2015) and forecast, with the Sen’s Slope method, at the end of 2nd and 3rd RBMP cycles (2021, 2027)

MP	Relative weight	Date range	NO_3_ (mg/L) in 2015	Trend (mg/L/year)	NO_3_ (mg/L) forecast 2021	NO_3_ (mg/L) forecast 2027
PC01-00	0	2001-2015	37.25	Not assessable	-	-
Pc03-02	8.33	2002-2015	40.90	+1.14	47.72	54.54
pc07-00	8.33	2001-2015	38.10	+1.33	46.09	54.08
pc15-01	8.33	2001-2015	30.90	+0.54	37.25	40.48
pc48-00	8.33	2001-2015	4.5	Not significant upward	-	-
pc56-00	8.33	2001-2015	42.05	+0.65	51.15	55.05
pc56-02	8.33	2001-2015	43.85	+0.62	46.70	50.40
pc56-08	0	2001-2015	45.40	Not assessable	-	-
pc69-00	8.33	2001-2015	49.20	+0.80	53.98	58.75
pc77-01	8.33	2007-2015	23.95	Not significant upward	-	-
pc81-00	8.33	2001-2015	71.45	+2.4	78.35	92.75
pc85-00	8.33	2001-2015	27.75	Not significant upward	-	-
pc87-01	8.33	2002-2015	13.00	Not significant upward	-	-
pc94-01	8.33	2003-2015	4.65	+0.17	5.36	6.37

As the protective measures are put in place, we can expect that a reversal (i.e. a statistically significant downward trend that follows a statistically significant upward trend) could be detected by the end of the next RBMP, in this case the 2nd cycle, hence in 2021. However, a preliminary impression of their effects on the upward trends might be appreciated using the currently available data, also because these measures might have been started prior to 2015 (Regione Emilia-Romagna 2005, 2006). To this end, the Pettitt test was applied to four MPs (PC03-02, PC07-00, PC69-00, PC81-00) of the six having a statistically and environmentally significant upward trend, using data collected up to 2018. For the remaining two MPs (PC56-00, PC56-02), the evaluation of trend reversal is not possible because the first has been discontinued and the second has not sufficient data to apply the Pettitt test (only 13 data available).

**Fig. 4:**
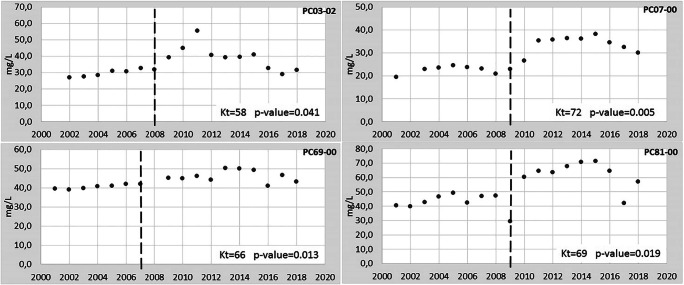
Analysis of the trend reversal for the nitrates by means of the Pettitt test. The vertical dashed line indicates the year identified as changing point by Pettitt test. Kt is the statistic of Pettitt test. If the *p* value ≤ 0.1, the test is statistically significant

The results of the Pettitt test applied to the time series of the four MPs are shown in Fig. 4. The Pettitt test was found to be able to detect changing points between two different slopes (including upward trends followed by stationary periods and vice versa) but the identified changing point is not always representative of a trend reversal (upward trend followed by downward trend). For this reason, we also applied the Mann-Kendall two-section test to the reversal verification, as described in “Trend reversal using the MK test”.

**Fig. 5:**
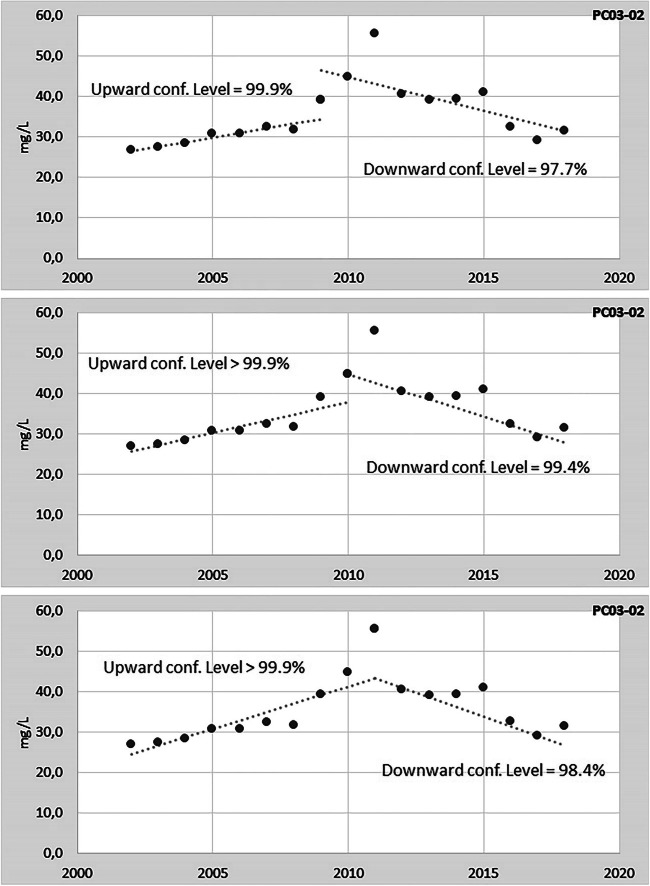
Analysis of the trend reversal by means of the Mann-Kendall two-section test for the nitrates at the monitoring point PC03-02 of the “Conoide Trebbia-Luretta” GWB. The dotted line represents the median slope of the trend, calculated according to the Sen’s method; the intercept is the median of all the intercepts

Applying the MK two-section test, three MPs do not show any significant trend reversal (PC07-00, PC69-00, PC81-00) so far, while the MP “PC03-02” shows a clear statistically significant trend reversal (Fig. 5). As for the MP “PC03-02”, the Mann-Kendall two-section test identifies three possible reversal points, in the years 2009, 2010 and 2011 (Fig. 5). In all three cases, the downward trend is statistically significant with the highest confidence level (99.4%) choosing 2010 as reversal year.

Based on the above, the comparison between the results of Pettitt test and MK two-section test shows that only one MP has a statistically significant trend reversal. Therefore, the reversal cannot yet be demonstrated at the GWB scale in 2018.

## Discussion

Gourcy et al. (2019) summarized the different methods used by 10 European Member States to analyse groundwater quality trends for the 1st RBMP. Mostly, the non-parametric Mann-Kendall test (and derivate Seasonal Kendall and Regional Kendall tests) or the ANOVA parametric test, in some cases combined with the LOESS smoother, were preferred (France, Romania, Hungary, Slovak Republic and the UK), while the parametric linear regression test was used by only one Member State (Poland) (Tab. S2). The application of parametric statistics assumes that sample data come from a population that can be adequately modelled by a probability distribution that has a fixed set of parameters; however, when few data are available, it is difficult to define a probability distribution. In addition, groundwater chemical data are rarely normally distributed (Edmunds and Shand 2004; UKTAG 2012). This reason, in addition to the lack of long time-series of groundwater quality data, led Italy among others to prefer non-parametric methods for a statistical evaluation of trends.

About the minimum number of years of monitoring used by European Member States for the trend assessment during the 1st RBMP cycle, a number between 5 and 10 years are reported by Gourcy et al. (2019), variable also in function of the monitoring frequency (yearly or half-annual) (Tab. S2). In Italy, a minimum of 8 years is required to ensure a statistically acceptable assessment, as with fewer data a possible trend can be masked by the white noise (Şen 2016; Hu et al. 2020) related to several factors, including natural variability or analytical uncertainty. A numerical explanation of this choice is provided in Appendix B, where the impact of the number of required years on the trend assessment is discussed showing differences arising from a dataset of 6 or 8 yearly observations. At the same time, eight yearly observations can be reasonably available in most situations. This minimum number of years is considered a compromise solution between the need to respond to the EU directives, the available data and the statistical robustness of the assessment.

An interpretation of “environmentally significant trend” which is largely shared among the Member States, converges towards an upward trend whose forecast exceeds the TV, or the starting point for trend reversal (75% of TV), at the end of the RBMP cycle. As for Italy, the exceedance of the 75% of TV at the end of the 2nd RBMP cycle or of the TV at the end of the 3rd RBMP cycle have been set as target for the identification of an environmentally significant trend. These limits have been chosen because their achievement precludes the possibility to define the GWB at good status (GWD).

As mentioned at “How to define a statistically and environmentally significant trend”, following the Italian Guidelines, a GWB has a significant upward trend if the trends observed at the single MPs affect more than 20% of the groundwater body (by surface extension or volume). However, the reference legislation does not clarify how to assess this exceedance. In Italy, as in many EU Member States, monitoring networks are heterogeneous and typically denser in the most impacted area (Collins et al. 2012); hence, it is difficult to define univocal rules for determining this exceedance. The Guidelines suggest to assign the same percentage by weight to each MP, both in the upward trend assessment and in the reversal trend assessment, or to assign a different percentage by weight based on the conceptual model when available. Another point to raise is that the Guidelines do not indicate how to manage data when one MP with an upward trend is dismissed and the monitoring network is reduced or modified. This event is not unusual in groundwater monitoring networks: MPs may collapse; the owner might deny sampling (frequently sampling points are private wells that are lent to the local monitoring agency). In these cases, an upward trend that should be tracked over time could be overlooked and the assessment at the GWB scale could be biased. The recommendation remains to select a robust monitoring network since the beginning. However, the significant and sustained upward trend that should be reversed and trend reversals to be demonstrated, regards the GWB scale. Therefore, we suggest to assess on the same spatial scale the reversal of trends that are significant at the GWB scale, using all the active MPs regardless of those used for the previous assessment. The percentage by weight of each point should be recalculated considering only the MPs that are still active.

Regarding the trend reversal analysis, in the case study discussed in this paper, we found that Pettitt test, although widely used to detect the change point in time series of climatic and hydrological variables (Serinaldi and Kilsby 2015; Verstraeten et al. 2006), seems less adequate to meet the requests of the GWD. The ranking statistics on which the Pettitt test is based makes it able to distinguish an upward (or downward) trend subset from a stationary subset (or vice versa) but seems less adapt to identify a pure reversal point, i.e. a shift from an upward trend to a downward trend, at least with so little data. This is particularly true when the changing point is located at the extremes of the distribution (Mallakpour and Villarini 2016). The MK two-section method that we propose seems more reliable in identifying a reversal point in quality time series. This is appreciable by comparing Figs. 4 and 5. For example, Pettitt test indicates 2008 as a changing point for PC03-02 (Fig. 4), while the graph clearly shows that in the following years (2009–2011) nitrates are higher than in 2008. On the other hand, for the same MP, the MK two-section test identifies three possible reversal years (2009, 2010, 2011) with the highest level of confidence for 2010 (Fig. 5). An important limitation of the MK two-section test is that, since at least 8 data are needed for each section and in this study the most recent data corresponds to 2018, the search for a significant downward trend must also use the data before 2015, i.e. before the end of the 1st RBMP. Therefore, the reversal point, by construction, lies between 2009 and 2011. In other words, this method has a pre-established, limited time window where the reversal year is located. In this specific case, this implies also that measures to reduce nitrates in groundwater were already in place at least in those years.

We concluded that the application of both methods of Pettitt test and the MK two-section test should be complemented by expert judgment to reinforce the results of the evaluation.

As affirmed also by Visser et al. (2009), statistical trend detection techniques are universally applicable, they require no additional costs of sampling if the quality of the available datasets is sufficient and the detection of trends is objective. However, the statistical approach should never overcome a conceptual understanding of the hydrogeological processes (Edmunds and Shand 2004; Lopez et al. 2015). Modifications in the sampling or analysis procedures might strongly influence the data sets (Wahlin and Grimvall 2008), uncertainty in the resulting data might mask real trends or produce false ones. Further, variations in the land use or in water management practices might result in upward trends related to impacts happened in the past and this could be difficult to distinguish without an appropriate knowledge of the groundwater body recent history. Finally, even the ongoing climatic variations could act on pollutants concentration and transport in the subsurface, eventually modifying actual trends (Lasagna et al. 2020). Further checks, applying also different methods (such as groundwater dating, deterministic flow and transport modelling), are always recommended.

## Conclusion

The Guidelines procedure can be applied to any groundwater body defined at risk in Europe for the assessment of upward trends of pollutants and identify trend reversals, finalized to the reporting to the European Commission in the RBMP, even when few chemical monitoring data are available. Notwithstanding, a sound and numerous information produced by the official monitoring networks is crucial, and the lack of data or poor sampling techniques cannot be overcome by sophisticated mathematical analysis techniques. In the case study presented, the MK two-section test seems more reliable than Pettitt test to identify a reversal point in quality time series. Although focused on the EU legislative framework, this procedure may be relevant for a wider context, allowing to individuate upward trend as early warning for contamination processes in an integrated water resource management context.

## Supplementary information

ESM 1 (DOCX 193 kb)

ESM 2 (XLSM 186 kb)

## Data Availability

All data and materials are publicly available.

## References

[CR1] Amirataee B, Zeinalzadeh K (2016). Trends analysis of quantitative and qualitative changes in groundwater with considering the autocorrelation coefficients in west of Lake Urmia, Iran. Environ Earth Sci.

[CR2] ARPAE (2020) https://www.arpae.it/elenchi_dinamici.asp?tipo=dati_acqua&idlivello=2020 (last access 23 April 2020)

[CR3] Collins A, Ohandja DG, Hoare D, Voulvoulis N (2012). Implementing the Water Framework Directive: a transition from established monitoring networks in England and Wales. Environ Sci Policy.

[CR4] Craig M, Daly D (2010). Methodology for establishing groundwater threshold values and the assessment of chemical and quantitative status of groundwater, including an assessment of pollution trends and trend reversal.

[CR5] Ducci D, Della Morte R, Mottola A, Onorati G, Pugliano G (2019). Nitrate trends in groundwater of the Campania region (southern Italy). Environ Sci Pollut Res.

[CR6] Edmunds WM, Shand P (2004). Geochemical baseline as basis for the European Groundwater Directive. Proc.WRI-11, Wanty and Seal II.

[CR7] EEA (2018) European waters assessment of status and pressures 2018. Report No 7/2018, European Environment Agency. 10.2800/303664

[CR8] Esterby SR (1996). Review of methods for the detection and estimation of trends with emphasis on water quality applications. Hydrol Process.

[CR9] European Commission (2009). Guidance on groundwater status and trend assessment. Guidance Document No. 18, Technical Report N. 026 – 2009.

[CR10] Giordano M (2009). Global groundwater? Issues and solutions. Annu Rev Environ Resour.

[CR11] Gourcy L., Lopez B. et al. (2019). Common implementation strategy for the Water Framework Directive and the Floods Directive. Technical report on groundwater quality trend and trend reversal assessment. Procedures applied by Member States for the first RBMP cycle, January 2019, Available on: https://circabc.europa.eu/ui/group/9ab5926d-bed4-4322-9aa7-9964bbe8312d/library/006b0646-6340-4233-8769-564fec15474a/details. Accessed 22 Apr 2020

[CR12] Grath J, Scheidleder A, Uhlig S, Weber K, Kralik M, Keimel T, Gruber D (2001). The EU Water Framework Directive: statistical aspects of the identification of groundwater pollution trends, and aggregation of monitoring results. Final Report.

[CR13] Griebler C, Avramov M, Hose G, Schröter M, Bonn A, Klotz S, Seppelt R, Baessler C (2019). Groundwater ecosystems and their services: current status and potential risks. Atlas of ecosystem services.

[CR14] Grima J, Luque-Espinar JA, Mejía JA, Rodríguez R (2015). Methodological approach for the analysis of groundwater quality in the framework of the Groundwater Directive. Environ Earth Sci.

[CR15] Helsel DR, Frans LM (2006). Regional Kendall test for trend. Environ Sci Technol.

[CR16] Hirsch RM, Alexander RB, Smith RA (1991). Selection of methods for the detection and estimation of trends in water quality. Water Resour Res.

[CR17] Hollander M, Wolfe DA (1973). Nonparametric statistical methods.

[CR18] Hollander M, Wolfe DA, Chicken E (2014). Nonparametric statistical methods.

[CR19] Hu Z, Liu S, Zhong G, Lin H, Zhou Z (2020). Modified Mann-Kendall trend test for hydrological time series under the scaling hypothesis and its application. Hydrol Sci J.

[CR20] Huang H, Wang Z, Xia F, Shang X, Liu Y, Zhang M, Dahlgren RA, Mei K (2017). Water quality trend and change-point analyses using integration of locally weighted polynomial regression and segmented regression. Environ Sci Pollut Res.

[CR21] ISPRA (2017) Environmental Data Yearbook - 2017. Ch.5 Inland Water Quality. 2017 Edition. Available on: https://annuario.isprambiente.it/pdf/environmental-data-yearbook. Accessed 18 Dec 2020

[CR22] ISPRA-CNR.IRSA (2017) Linee guida per la valutazione delle tendenze ascendenti e d’inversione degli inquinanti nelle acque sotterranee (DM 6 luglio 2016) (in Italian). ISPRA, Manuali e Linee Guida 161/2017, ISBN 978-88-448-0844-0.

[CR23] ISTAT (2017) Censimento delle acque per uso civile (in Italian). ISTAT Report. Roma. Available on: https://www.istat.it/it/files//2017/12/Report-Censimento-acque.pdf (accessed on 3 April 2020)

[CR24] Kendall MG (1975). Rank Correlation Methods.

[CR25] Lasagna M, Ducci D, Sellerino M, Mancini S, De Luca DA (2020). Meteorological variability and groundwater quality: examples in different hydrogeological settings. Water.

[CR26] Lopez B, Baran N, Buorgine B (2015). An innovative procedure to assess multi-scale temporal trends in groundwater quality: example of the nitrate in the Seine–Normandy basin, France. J Hydrol.

[CR27] Mallakpour I, Villarini G (2016). A simulation study to examine the sensitivity of the Pettitt test to detect abrupt changes in mean. Hydrol Sci J.

[CR28] Mann HB (1945) Nonparametric tests against trend. Econometrica:245–259

[CR29] Martin-Ortega J (2012). Economic prescriptions and policy applications in the implementation of the European Water Framework Directive. Environ Sci Policy.

[CR30] Oliva F, Vegas E, Vicit S, Garrido T, Fraile J, Munné A, Munné A, Ginebreda A, Prat N (2016). Trend assessment for groundwater pollutants: a brief review and some remarks. Experiences from ground, coastal and transitional water quality monitoring: the EU Water Framework Directive implementation in the Catalan River Basin District (Part II).

[CR31] Onorati G, Di Meo T, Bussettini M, Fabiani C, Farrace MG, Fava A, Ferronato A, Mion F, Marchetti G, Martinelli A, Mazzoni M (2006). Groundwater quality monitoring in Italy for the implementation of the EU water framework directive. Phys Chem Earth.

[CR32] Pastor AV, Palazzo A, Havlik P, Biemans H, Wada Y, Obersteiner M, Kabat P, Ludwig F (2019). The global nexus of food–trade–water sustaining environmental flows by 2050. Nat Sustain.

[CR33] Pettitt AN (1979). A non-parametric approach to the change-point problem. Appl Stat.

[CR34] Qiu J, Zipper SC, Motew M, Booth EG, Kucharik CJ, Loheide SP (2019). Nonlinear groundwater influence on biophysical indicators of ecosystem services. Nat Sustain.

[CR35] Regione Emilia-Romagna (2005).Piano di Tutela delle Acque. Deliberazione dell’Assemblea Legislativa della Regione Emilia-Romagna n. 40 del 21/12/2005 (in Italian). Available on: http://ambiente.regione.emilia-romagna.it/it/acque/temi/piano-di-tutela-delle-acque (accessed on 3 April 2020).

[CR36] Regione Emilia-Romagna (2006) Strategic contents of policies in the Water Protection Plan of Emilia-Romagna Region. Available on: http://ambiente.regione.emilia-romagna.it/it/acque/approfondimenti/documenti/piano-di-tutela-delle-acque/water-protection-plan-strategic-contents-of-policies/piano_tutela_acque_english.pdf (accessed on 3 April 2020).

[CR37] Regione Emilia-Romagna (2010) Approvazione delle attività della Regione Emilia-Romagna riguardanti l’implementazione della Direttiva 2000/60/CE ai fini della redazione e adozione dei Piani di Gestione dei Distretti idrografici Padano, Appennino settentrionale e Appennino centrale. Delibera di Giunta della Regione Emilia-Romagna n. 350 del 8/02/2010 (in Italian). Available on: http://ambiente.regione.emilia-romagna.it/acque/temi/piani%20di%20gestione (accessed on 3 April 2020)

[CR38] Salmi T, Määttä A, Anttila P, Ruoho-Airola T, Amnell T (2002). Detecting trends of annual values of atmospheric pollutants by the Mann-Kendall test and Sen’s slope estimates – the excel template application MAKESENS. Pubblications on Air Quality, 31.

[CR39] Scheidleder A., Grath J., Winkler G., Stärk U., Koreimann C., Gmeiner C., Nixon S., Casillas J., Gravesen P., Leonard J. and Elvira M. (1999). Groundwater quality and quantity in Europe. Environmental Assessment Report n.3. European Environment Agency, Copenhagen, 1999

[CR40] Sen PK (1968). Estimates of the regression coefficient based on Kendall's tau. J Am Stat Assoc.

[CR41] Şen Z (2016). Hydrological trend analysis with innovative and over-whitening procedures. Hydrol Sci J.

[CR42] Serinaldi F, Kilsby CG (2015). The importance of prewhitening in change point analysis under persistence. Stoch Env Res Risk A.

[CR43] UKTAG (2012) Groundwater trend assessment. UK Technical Advisory Group on the Water Framework Directive, 7 pp, February 2012. Available on: http://www.wfduk.org/resources%20/groundwater-trend-assessment. Accessed 22 Apr 2020

[CR44] Urresti-Estala B, Jiménez Gavilán P, Vadillo Pérez I, Carrasco Cantos F (2016). Assessment of hydrochemical trends in the highly anthropised Guadalhorce River basin (southern Spain) in terms of compliance with the European groundwater directive for 2015. Environ Sci Pollut Res.

[CR45] Verstraeten G, Poesen J, Demaree G, Salles C (2006) Long-term (105 years) variability in rain erosivity as derived from 10-min rainfall depth data for Ukkel (Brussels, Belgium): implications for assessing soil erosion rates. J Geophys Res 111. 10.1029/2006JD007169

[CR46] Visser A, Dudus I, Broers HP, Brouyère S, Korcz M, Orban P, Goderniaux P, Battle-Aguilar J, Surdyk N, Amraoui N, Job H, Pinault JL, Bierkens M (2009). Comparison of methods for the detection and extrapolation of trends in groundwater quality. J Environ Monit.

[CR47] Wahlin K, Grimvall A (2008). Uncertainty in water quality data and its implications for trend detection: lessons from Swedish environmental data. Environ Sci Policy.

[CR48] Zhang Q, Liu C, Xu C, Xu Y, Jiang T (2006). Observed trends of annual maximum water level and streamflow during past 130 years in the Yangtze River basin, China. J Hydrol.

