# Supplementary material for: Groundwater quality trend and trend reversal assessment in the European Water Framework Directive context: an example with nitrates in Italy
Source: Environ Sci Pollut Res Int. 2021 Jan 7;28(17):22092–104. doi: 10.1007/s11356-020-11998-0 (PMC8106612; doi:10.1007/s11356-020-11998-0)
Supplement: Supplementary file 1 — (DOCX 193 kb) [file 11356_2020_11998_MOESM1_ESM.docx]

**Supplementary materials**

|  | **NO_3_ (mg/L)** | | | | | | | | | | | | | | | | | |
| --- | --- | --- | --- | --- | --- | --- | --- | --- | --- | --- | --- | --- | --- | --- | --- | --- | --- | --- |
| **MS** | **2001** | **2002** | **2003** | **2004** | **2005** | **2006** | **2007** | **2008** | **2009** | **2010** | **2011** | **2012** | **2013** | **2014** | **2015** | **2016** | **2017** | **2018** |
| **PC01-00** | 48.85 | 48.30 | 48 | 47.30 | 47.00 | 48.90 | 49.05 | 49.3 |  |  |  |  | 32.25 | 5.10 | 37.25 | 70.00 | 53.00 | 71.00 |
| **PC03-02** |  | 26.80 | 27.4 | 28.40 | 30.80 | 30.70 | 32.45 | 31.75 | 39.15 | 44.75 | 55.45 | 40.45 | 39.00 | 39.35 | 40.90 | 32.50 | 29.00 | 31.50 |
| **PC07-00** | 19.30 |  | 22.95 | 23.45 | 24.50 | 23.65 | 23.10 | 20.85 | 22.75 | 26.55 | 35.35 | 35.60 | 36.30 | 36.05 | 38.10 | 34.50 | 32.50 | 30.00 |
| **PC15-01** | 24.45 | 25.55 | 27.55 | 27.65 | 28.55 | 28.55 | 28.05 | 26.55 | 31.25 | 32.55 | 34.15 | 32.40 | 31.45 | 31.90 | 30.90 | 31.50 | 29.50 | 28.00 |
| **PC48-00** | 3.80 | 3.05 | 3.1 | 4.15 | 4.30 | 6.50 | 6.25 | 7.90 | 6.10 | 7.85 | 7.35 | 5.55 | 4.10 | 4.70 | 4.50 | 4.50 | 4.50 | 5.50 |
| **PC56-00** | 38.15 | 38.15 | 39.15 | 40.05 | 40.75 | 39.60 | 38.65 | 39.50 | 41.6 | 44.00 | 47.40 | 41.80 | 49.50 | 50.50 | 42.05 |  |  |  |
| **PC56-02** |  |  |  |  |  | 38.40 | 37.35 | 38.75 | 40.15 | 39.25 | 42.70 | 40.30 | 43.90 | 45.20 | 43.85 | 45.00 | 45.00 | 44.00 |
| **PC56-08** | 42.10 | 42.2 | 42.70 | 45.00 | 46.00 | 45.30 | 45.50 | 46.80 |  |  |  |  | 60.75 | 68.50 | 45.40 | 58.00 | 56.50 | 56.50 |
| **PC69-00** | 39.30 | 38.85 | 39.65 | 40.70 | 40.80 | 41.85 | 41.85 |  | 45.00 | 44.70 | 46.05 | 44.15 | 50.05 | 49.80 | 49.20 | 41.00 | 46.50 | 43.00 |
| **PC77-01** |  |  |  |  |  |  | 7.70 | 6.55 | 26.15 | 31.30 | 33.70 | 5.45 | 25.10 | 11.35 | 23.95 | 9.50 | 9.50 | 10.00 |
| **PC81-00** | 40.45 | 39.65 | 42.60 | 46.60 | 49.05 | 42.35 | 47.05 | 47.15 | 29.25 | 60.30 | 64.60 | 63.50 | 67.85 | 70.65 | 71.45 | 64.50 | 42.00 | 59.00 |
| **PC85-00** | 28.30 | 28.9 | 26.90 | 30.80 | 32.50 | 31.65 | 28.10 | 26.60 | 31.05 | 34.20 | 34.50 | 32.00 | 29.05 | 31.15 | 27.75 | 26.00 | 26.50 | 24.00 |
| **PC87-01** |  | 14.7 | 12.35 | 15.65 | 15.85 | 15.85 | 16.4 | 16.10 | 17.80 | 13.95 | 15.45 | 15.80 | 14.35 | 16.35 | 13.00 | 15.00 | 14.50 | 12.00 |
| **PC94-01** |  |  | 2.30 | 3.25 | 2.50 | 2.65 | 3.00 | 1.95 | 3.10 | 3.50 | 2.15 | 1.80 | 4.60 | 5.05 | 4.65 | 2.50 | 1.25 | 1.5 |

Table S1. Annual concentration of NO_3_ (mg/L) in the MPs of “*Conoide Trebbia-Luretta*” GWB between 2001 and 2018 (average of two seasonal measures)

| Member State | Statistical method for trend determination | Minimum number of years of monitoring results | When a trend is environmentally significant |
| --- | --- | --- | --- |
| Austria | ANOVA | - 8 years for annual data - 6 years for half-annual and quarterly data | When the trend line exceeds the starting point for trend reversal |
| Czech Republic | - Two-section test for pollutants and MPs >18 and at least 10 years; - Linear regression for pollutants and MPs>9 and at least 6 years; - Comparison of two means for pollutants and MPs with at least 6 years (but results not significant) | - 6 years (more than 9 results) for significant trend - 4 years, but the results are not yet significant | Trend is significant if the threshold value is exceeded in 2015; it is potentially significant if the TV is exceeded in 2017 |
| Denmark | Comparison between the mean values of each period | None minimum number of years is provided, but it is required that chemical analysis have been available for each of the two period | When MPs have a strongly increasing parameter values (>10%). |
| France | - Kendall regional at GWB level - Mann-Kendall test at monitoring point | None minimum number of years is required, but at least 10 monitoring data available after the 1996 | If the 40% of TV is reached at the end of the WFD cycle |
| Hungary | Mann-Kendall test | 5 years | When the trend line exceeds the starting point for trend reversal within the examined time period and when the trend line exceeds the TV in the next two river basin cycles |
| Italy | Mann-Kendall test | 8 years | When the predicted concentration exceeds the 75% of TV at the end of 2^nd^ RBMP cycle and it exceeds the TV at the end of the 3^rd^ RBMP cycle |
| Poland | Linear regression | - 8 years for annual data - 6 years for half-annual and quarterly data | When the predicted concentration exceeds the starting point for the trend reversal |
| Romania | ANOVA | 8 years for annual data | No environmental significance was defined for trends |
| Slovak Republic | - Mann-Kendall test in each time series; - ANOVA for normal distributed data | 6 years | The mean of 2 last yearly mean concentrations ≥ 75% of the TV and/or the predicted concentration for 2021 ≥ TV. |
| United Kingdom | - Seasonal Kendall test when there are sufficient data within a given year; - Sen’s method when data are variable of insufficient - Linear regression analysis is carried out as a further test for trends | - 6-10 years for anthropogenic trends - At least 6 years for natural trends. At least 4 years with minimum 10 results in England and Wales | When the predicted concentration at the end of the two river basin cycles exceeds the TV |

Table S2. Comparison of methods and assumptions used by Member States for the statistically and environmentally significant trend assessment (data from Gourcy et al. 2019).

# **Appendix A**

A spreadsheet (MP_trend_Analysis.xlsm) has been developed in Excel© in order to assist operators and stakeholders in the application of the Italian Guidelines for the trend analysis. This spreadsheet is available as supplementary material. This tool allows the identification of statistically significant trends in water quality data sets on a single monitoring point. Specific values of concentration threshold, LOQ and the significance threshold for the Mann-Kendall test should be entered in the spreadsheet by the user. In the first sheet (labelled “Data”), the available monitoring data for a particular compound are averaged on a yearly basis; thus, the obtained average values are automatically plotted onto a graph (concentration versus time); before the averaging calculation, values < LOQ are automatically transformed into LOQ/2. In the second sheet (“MK – Pettitt”), the non parametric Mann-Kendall test is applied on the annual values. Since the annual dataset numerosity is always < 40, the *S* statistics from Hollander et al. (2014, 1973 implemented in the sheet labelled “Table”) for the one-tail test was implemented. Confidence values, not present in the original table, were calculated by interpolation. The confidence for a one-tail distribution is calculated and the null hypothesis is verified against the assigned threshold.

Then, the slope of the trend is computed with the non-parametric Sen’s method (Sen 1968). The theoretical scenarios of the concentration at 2021 and 2027 correspond to the date of assessment (2015) plus one (6 years) and two (12 years) River Basin Management Plan cycles of observation. They are calculated using the slope and the intercept with the y axis, both obtained as the median values for all possible couple of points, as required by the method. This calculation differs from what the Guidelines specify, i.e. it calculates the future values by adding the increases (given by the trend multiplied by the number of years) to the last observed concentration value. Further, the theoretical date when the 75% and 100% of the threshold will be reached is supplied.

Further down in the same sheet (“MK – Pettitt”) at the rows 72-98, the Pettitt test is applied. The calculation is performed using a macro procedure activated by a button. The underlying VBA code is:

Sub Pulsante1_Click()

Range("D95:Y95") = ""

For t = 1 To (Range("J24") - 1)

somma = 0

For i = 1 To t

somma1 = 0

For j = t + 1 To Range("J24")

somma1 = somma1 + Cells(71 + j, 3 + i)

Next j

somma = somma + somma1

Next i

Cells(95, t + 3) = somma

Next t

End Sub

In the sheets “MK two-section_1” and “MK two-section_2” the Mann –Kendall two-section test is applied. In the first one (“MK two-section_1”), the user should enter the data for the first (ascending) section (until the year that the user wants to test as possible reversal point). In the second one (“MK two-section_2”), the user should enter the data for the second (descending) section (starting with the year that the user wants to test as reversal point). In the “MK two-section_2”, a chart displays all the available data for both the first and second sections.

The spreadsheet MP_trend_Analysis.xlsm is available as supplementary material.

# **Appendix B**

We analyse the dependence of the Kendall distribution S on the number of observations (6 and 8) and on the possible white noise to measure random variability comparing four groups of 100.000 time series each, randomly generated as follows:

1. 100.000 steady state time series of 6 (8) yearly observations following a normal distribution with mean = 150 (e.g. 150 mg/l of a given chemical compound) and standard deviation = 20 (i.e. the associated measurement precision).
2. To each time series of 6 (8)observations, a trend of 6 mg/l/year is imposed
3. The S statistics is computed on the four groups of 100.000 time series generated according to points 1 and 2 (that is, two series with trend and two series without trend, of 6 and 8 elements respectively). The obtained empirical cumulative distribution function are shown in figure B1.


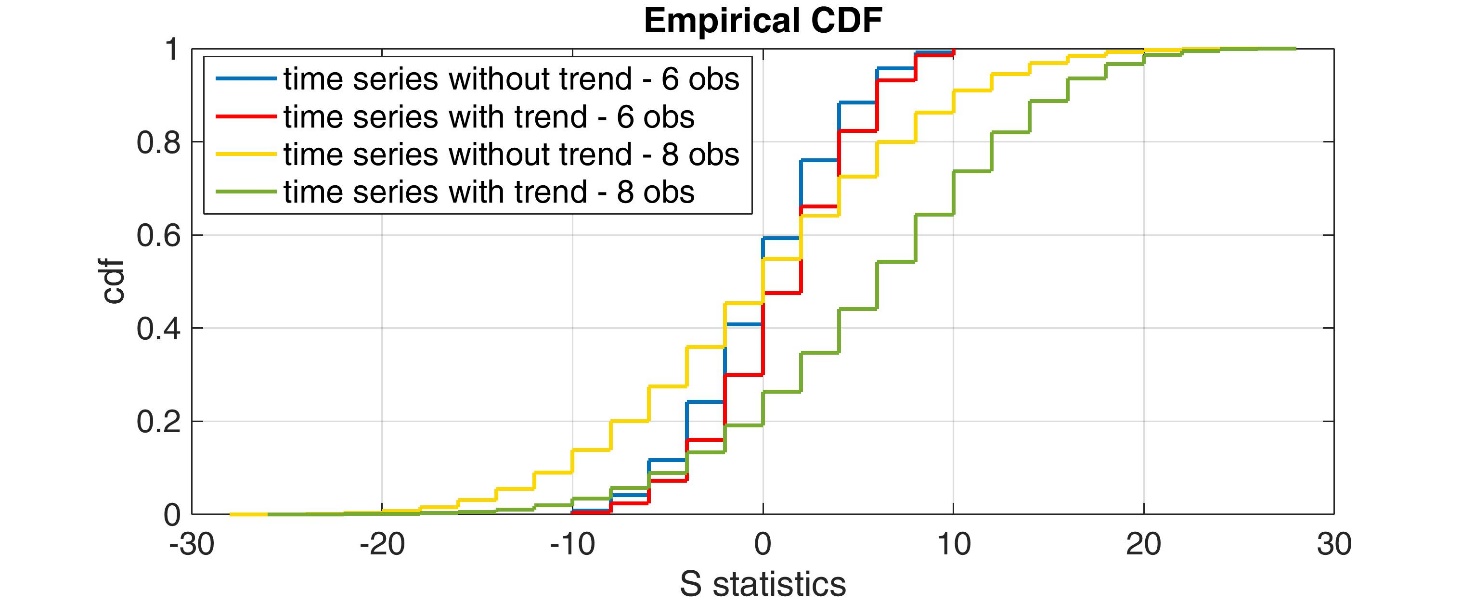


Figure B1. Sample Cumulative Distribution Function of the Mann-Kendall statistics S as a function of the number of observations and on the presence/lack of trend

As shown in figure B1, the CDF of the MK statistic for the time series of 6 observations with or without trend (blue and red lines) nearly overlap between -10 and +10. With 8 yearly observations the S statistics for the series with trend (green) or without trend (yellow) have a much wider range (-30, +30) and they clearly differ of about 6 as an average. The reported example may vary as a function of the imposed white noise and trend, however it shows that actual trends can be masked by the white noise if the observations are too few and the trend may remain unrevealed.
